# *GRASP Integrated 3D Plotter*: *GRIP*

**DOI:** 10.1107/S1600576724010379

**Published:** 2024-11-26

**Authors:** Paul M. Neves, Jonathan S. White

**Affiliations:** a Massachusetts Institute of Technology, USA; b Paul Scherrer Institut Switzerland; Argonne National Laboratory, USA

**Keywords:** small-angle neutron scattering, small-angle neutron diffraction, software, data analysis, data visualization, *GRIP*, *GRASP*

## Abstract

In research on mesoscale structure and correlations, small-angle neutron scattering (SANS) is increasingly being employed to map fully three-dimensional distributions of scattered intensity at low momentum transfer. While traditionally SANS experiments and data analysis methods are designed to prioritize the determination of salient information in only one or two dimensions, the trend towards volumetric intensity mapping experiments calls for new software tools to assist with analyzing the resulting datasets. In this paper, we describe the development of a new software module, the *GRASP Integrated 3D Plotter* (*GRIP*). *GRIP* adds numerous features to *GRASP*, a widely used SANS analysis program that was written in MATLAB and developed at the Institut Laue–Langevin, France. The *GRIP* module provides multiple methods of three-dimensional SANS data visualization and new abilities to perform 1D and 2D cuts in various momentum-space coordinate systems, including reciprocal lattice units relevant for single-crystal studies. *GRIP* also includes the ability to fit diffraction peaks to a fully three-dimensional ellipsoidal Gaussian function to extract peak parameters including peak intensity, location and width, as well as a built-in calculator for estimating the resolution-deconvolved 3D coherence lengths in a sample. *GRIP* thus represents a significant addition to *GRASP* which extends the utility and application of SANS. Valuable advantages are provided, in particular, for ‘small-angle neutron diffraction’ studies of mesoscale correlations in single crystals, such as those due to incommensurate magnetic spin textures like spirals and topological skyrmion lattices.

## Abstract 2

This article describes the implementation of *GRIP* as a module of *GRASP*, enabling the fully three-dimensional visualization and analysis of data collected on small-angle neutron scattering instruments.

## 1. Introduction

Small-angle neutron scattering (SANS) is a well established and versatile technique (Jeffries *et al.*, 2021 ▸) providing pivotal insights in the research fields of biological systems, polymers, engineering, nanoparticles and micromagnetism (Mühlbauer *et al.*, 2019 ▸; Jacques & Trewhella, 2010 ▸; Koch *et al.*, 2003 ▸; Schmidt, 1991 ▸; Chen, 1986 ▸; Gabel *et al.*, 2002 ▸; Schmatz *et al.*, 1974 ▸; Bunjes & Unruh, 2007 ▸; Blazek & Gilbert, 2011 ▸; Marshall & Lowde, 1968 ▸; Gebel & Diat, 2005 ▸; Honecker *et al.*, 2022 ▸), to name a few. The technique is optimized for the study of structure and correlations characterized by real-space length scales in the ∼1–400 nm range. In recent years in particular, SANS has proved invaluable in the investigation of a growing number of magnetic materials found to host complex magnetism such as magnetic incommensurately modulating spiral and skyrmion phases (Mühlbauer *et al.*, 2009 ▸; Mühlbauer *et al.*, 2019 ▸; Tokura & Kanazawa, 2021 ▸). Skyrmions themselves are nanoscale magnetic whirl-like structures with topological properties and real-space length scales that vary from a few to a few hundred nanometres. As they typically form a two-dimensional hexagonal lattice inside a host crystal, the distribution of the associated diffraction signals in momentum space from these skyrmion lattices makes them ripe for exploration and characterization by SANS. The first discovery by SANS of skyrmions was in the chiral cubic helimagnet MnSi in 2009 (Mühlbauer *et al.*, 2009 ▸; Neubauer *et al.*, 2009 ▸; Yu *et al.*, 2010 ▸); skyrmions and their lattice structures have most recently been discovered in frustrated magnets with strong Ruderman–Kittel–Kasuya–Yosida (RKKY) interactions (Kurumaji *et al.*, 2019 ▸; Hirschberger *et al.*, 2019 ▸), with SANS studies again making notable contributions (Takagi *et al.*, 2022 ▸; Singh *et al.*, 2023 ▸). Beyond two-dimensional skyrmion lattices, three-dimensional magnetic textures such as hedgehog lattices (Tanigaki *et al.*, 2015 ▸; Fujishiro *et al.*, 2019 ▸; Ishiwata *et al.*, 2020 ▸; Kanazawa *et al.*, 2020 ▸) have also been revealed using SANS, extending the scope of magnetic textures found in nature that may be potentially exploited for industrial use (Fert *et al.*, 2017 ▸).

The developing interest in applying SANS instruments for studying diverse types of incommensurate magnetic order marks the evolution towards a need for comprehensive analysis of three-dimensional datasets. Traditionally SANS data analysis software allows the user to perform an analysis over either one or two dimensions within the *q*_*x*_-*q*_*y*_ plane of the two-dimensional detector (Pedersen, 1997 ▸). The *q*_*z*_ dimension (parallel to the incident neutron beam) is otherwise neglected or integrated over, and in many studies, for instance on biological and soft matter systems, only |**q**| is considered. However, in systems with sharply peaked structure factors or single-crystalline materials, important information is con­tained in the *q*_*z*_ dimension: namely the three-dimensionality of propagation vectors **q** of incommensurate magnetic structures, information about mosaicity of single-crystal samples and three-dimensional correlation lengths. The emergence of numerous single-crystalline materials hosting magnetic textures spanning a wide distribution in momentum space strongly motivates the need for SANS software tools which allow visualization, analysis and interpretation of fully three-dimensional diffraction data, thus consolidating a branch of the technique we term small-angle neutron diffraction (SAND).

To date, SANS software tools have provided only limited support for the detailed analysis of the full three-dimensional scattering that can be collected in standard SAND measurements (*i.e.* ‘rocking curve’ measurements), where multi-detector intensity data are collected over a range of discrete sample rotation angles with respect to the incoming neutron beam. Notably, the SANS data reduction and analysis software *GRASP*, written in MATLAB (The MathWorks Inc., Natick, MA, USA) and developed at the Institut Laue–Langevin (ILL), Grenoble, France (Dewhurst, 2023 ▸), provides a platform for analysis of SAND data obtained over a range of rocking angles, as well as the ability to analyze data both within the *q*_*x*_-*q*_*y*_ detector plane and as a function of rocking angle. However, the standard analysis tools offered by *GRASP* neither calculate *q*_*z*_ nor transform the data from the laboratory frame into the sample’s momentum space, and rely on integration over one or more directions of a three-dimensional array of the accumulated two-dimensional SANS detector data. Additionally, there are no tools that allow the user to either visualize or analyze the entire three-dimensional dataset as a single entity of volumetric data in the sample reference frame.

Here, we describe the development of a user module for *GRASP* that provides the capability for the operator to both visualize and analyze an entire three-dimensional SAND dataset. This user module, called *GRASP Integrated 3D Plotter* or *GRIP*, harnesses much of the flexibility of *GRASP*, making it immediately compatible with data collected at numerous SANS instruments around the world, as well as including support for modern multi-panel SANS detectors and polarization analysis. *GRIP* introduces several methods of plotting three-dimensional SAND datasets, the ability to make user-defined one- and two-dimensional cuts in a variety of coordinate systems, and a SAND calculator to aid in the planning of experiments. Beyond this, *GRIP* elevates the capability of *GRASP* to a full 3D analysis of SAND data through (i) plotting the three-dimensional SAND dataset in reciprocal lattice units, (ii) three-dimensional fitting of diffraction peaks to extract 3D **q**-vector lengths and orientations, diffraction intensities, and correlation lengths, and (iii) a built-in resolution calculator. Additionally, the processed datasets produced by *GRIP* are made available to users using the MATLAB-code version as a global data structure in the MATLAB workspace. The current version of *GRIP* was developed in MATLAB version R2023b with *GRASP* version 10.27f. *GRIP* is included as part of *GRASP*, either as source MATLAB code or as a standalone executable, and is distributed freely by the ILL at https://www.ill.fr/grasp.

## 2. Formalism

### 2.1. Coordinate systems

**Figure 1:**
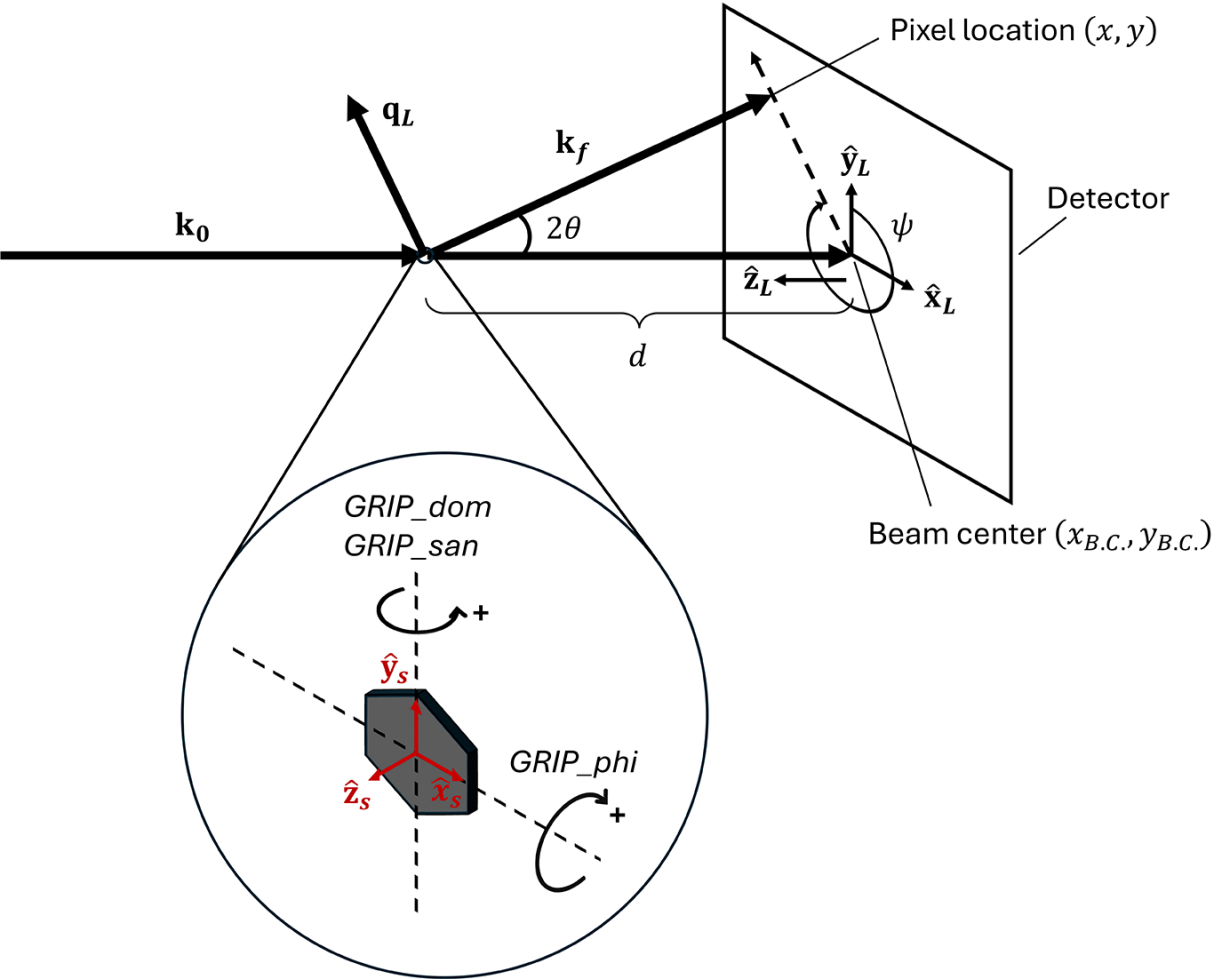
Diagram of a typical SANS experiment geometry at a continuous source, and angle/axis coordinates used by *GRIP*. An incident collimated and monochromated neutron beam comes from the left with wavevector **k**_0_. The sample is indicated by a gray hexagonal prism with local sample coordinates 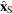, 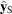, 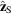 in red. The sense of rotation of the goniometer angles GRIP_dom, GRIP_san and GRIP_phi is indicated about the axes shown as dashed lines. Outgoing scattered neutrons at wavevector **k**_f_ impinge upon a detector a distance *d* (several metres, not to scale) away. The laboratory coordinate system 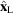, 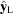, 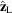 is indicated on the detector, along with the Bragg angle 2θ and the azimuthal angle ψ.

In order to develop the full three-dimensional treatment of SAND data, it is important to first describe the coordinate systems and their relationships. The geometry of a standard SANS experiment is depicted in Fig. 1 ▸. A collimated monochromatic neutron beam is transmitted through a sample, and then the scattered beam is detected on a 2D detector a distance *d* behind the sample. We define a dimensionless laboratory coordinate system with an origin in the plane of the detector where 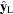 is vertical, 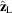 is anti-parallel to the incident neutron beam and 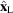 completes the right-handed coordinate system. The incident neutron wavevector 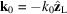, where the magnitude *k*_0_ is related to the neutron wavelength λ by *k*_0_ = 2π/λ. Since we consider only standard elastic scattering processes [neither inelastic SANS nor scattering processes that lead to noticeable changes in neutron kinetic energy (*e.g.* Kealey *et al.*, 2001 ▸; Rastovski *et al.*, 2013 ▸) are considered or implemented in *GRIP*], the outgoing neutron wavevector 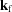 of magnitude *k*_0_ forms an angle 2θ with 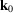 and an azimuthal angle ψ in the *x*-*y* plane. If a neutron is detected at a position (*x*, *y*, 0) on the detector, and the transmitted beam is centered at (*x*_BC_, *y*_BC_, 0), then 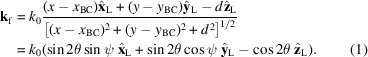
The scattering wavevector in the laboratory coordinate system 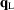 is then defined as 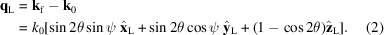


Prior to *GRIP*, this equation represented the extent to which *GRASP* handled both geometric angles and the momentum-space coordinate system, with the relevant values generated by the built-in *GRASP* function build_q_matrix.m. Combining these values with an understanding of the geometry of the rotation axes, the *GRIP* module maps these data to a three-dimensional reciprocal space in the reference frame of the sample. In a standard experiment, the sample and cryostat may be rocked together, first by a rotation about the vertical direction, which we refer to as *san*, and then on that goniometer by a rotation about a horizontal axis parallel to 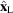 when *san* = 0, referred to as ϕ. Then inside the cryostat the sample stick can be rotated about an axis that is parallel to 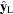 when ϕ is zero, a rotation that we refer to as *dom*. (The names of angles *san*, ϕ and *dom*, respectively, correspond to those commonly called ω, χ and ϕ on four-circle diffractometers.) The combined effect of these independent rotational degrees of freedom can be represented by the application of three consecutive rotation matrices: 
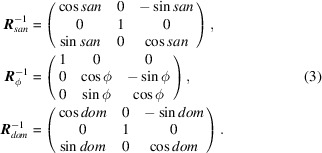
These create a master rotation matrix 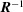,
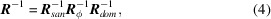
that transforms the scattering vector in the laboratory coordinate system 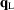 into the (not yet aligned) sample coordinate system 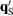 as 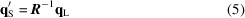
when 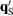 and 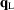 are represented as row vectors. A standard SAND experiment consists of rocking over one or several angles and taking exposures on the 2D detector at many discrete, consecutive angles. In this way, a three-dimensional volume of reciprocal space is mapped out. To make any of the *GRASP*-supported SANS instruments compatible with *GRIP*, one need only add a mapping of the instrument goniometer angles to the GRIP_dom, GRIP_san and/or GRIP_phi variables within the instrument’s specific pre-existing *GRASP* configuration file.

*GRIP* provides further support for correcting any mis­align­ment of the sample with respect to the zeroed goniometer positions. Here three additional rotation matrices 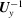, 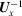 and 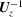 correct for misalignment of the sample rotation in *dom*, tilt in ϕ and azimuthal alignment, respectively. These are written as
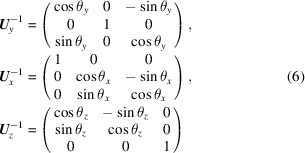
for angular sample misalignments θ_*y*_, θ_*x*_ and θ_*z*_. The complete transformation matrix from the laboratory 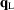 to an aligned sample reciprocal space 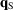 is thus 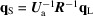 with 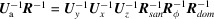.

**Figure 2:**
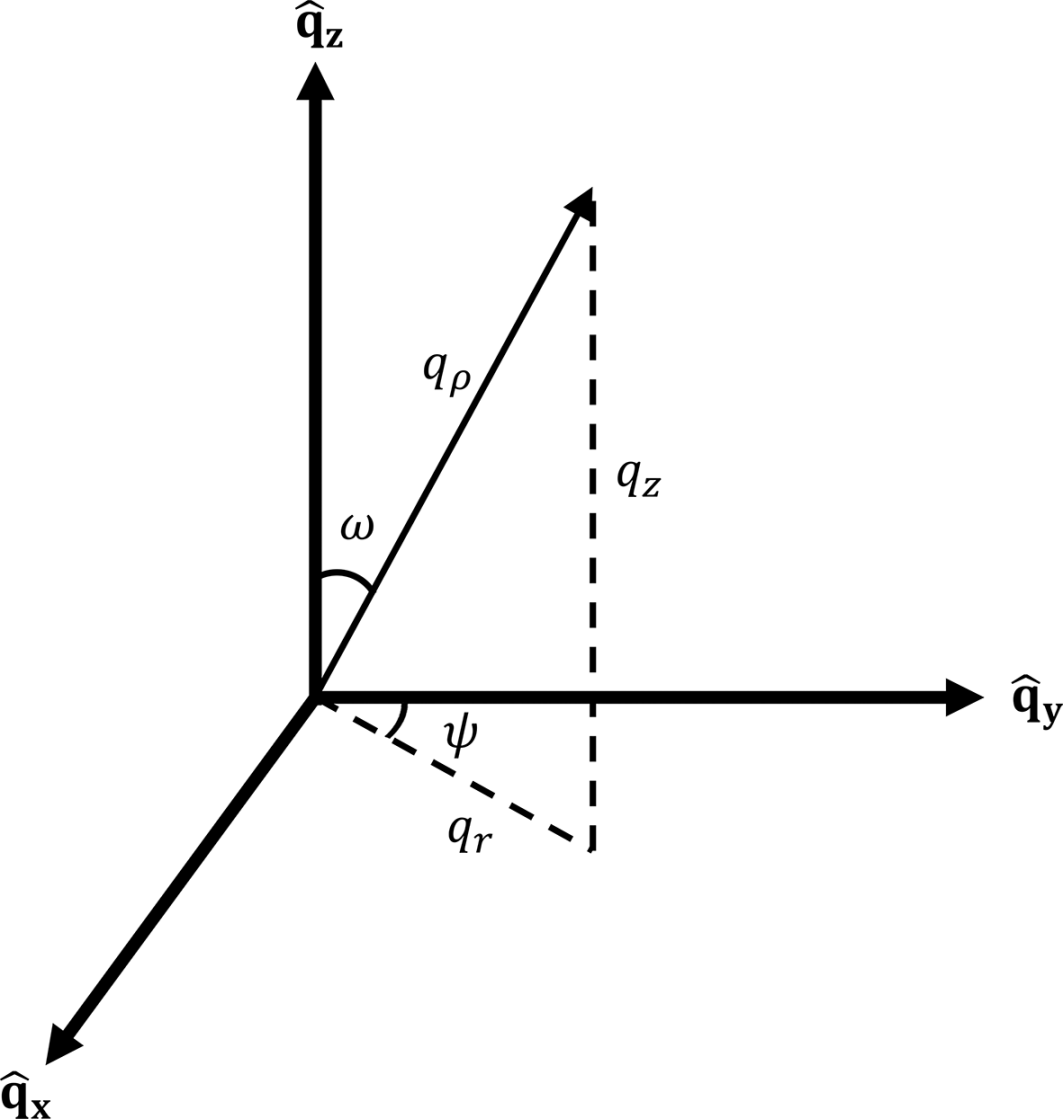
Relationship between Cartesian (*q*_*x*_, *q*_*y*_, *q*_*z*_), cylindrical (*q*_*r*_, ψ, *q*_*z*_) and spherical (*q*_ρ_, ψ, ω) coordinate systems used by *GRIP*.

*GRIP* also supports several other coordinate systems which can aid in various common types of data cuts, visualizations and analysis that the users may want to perform. A cylindrical coordinate system is provided by 
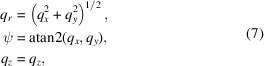
where atan2 is the four-quadrant arctangent function returning in units of degrees such that 0° ≤ atan2(*y*, *x*) ≤ 360°. This coordinate system is particularly useful, for example, when analyzing hexagonal/square lattice structures, or other cases where a magnetic field creates a planar or cylindrically symmetric structure, or where a user observes an azimuthal distribution of intensity as a function of *q*_*z*_ or *q*_*r*_. Additionally, a spherical coordinate system is provided by 
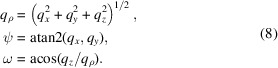
This coordinate system is particularly convenient for obtaining the true magnitude of 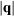 that includes all three dimensions, and for resolution function calculations, since the principal axes of the instrument resolution function lie along *q*_ρ_, ψ and ω when the rocking axis is perpendicular to 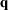. The relationship between the Cartesian, cylindrical and spherical coordinate systems is depicted in Fig. 2 ▸.

Also of key importance for interpreting SAND data from single-crystalline samples is the ability to transform the data into the reciprocal lattice unit (RLU) coordinate system of the sample. This transformation can be accomplished by 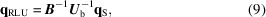
where 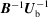 is a matrix which transforms coordinates in momentum space into reciprocal lattice units (Busing & Levy, 1967 ▸; Arnold *et al.*, 2014 ▸). In SANS, it is often not possible to directly access nuclear Bragg peaks, so alignment must be done either *ex situ* or with (in)commensurate superlattice peaks of known propagation direction. For this reason, we separate the sample misalignment correction matrix 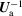 from the overall 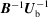 matrix for convenience. 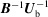 can be generated from a set of three points with known coordinates in both 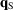 and 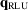 as 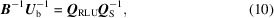
where 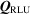 and 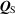 are 3 × 3 matrices made of three columns of three vectors of 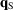 and 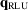, respectively. The 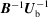 matrix can also be generated from two known crystallographic directions if the lattice constants are known (Busing & Levy, 1967 ▸; Arnold *et al.*, 2014 ▸). Both methods have been implemented in *GRIP*.

To aid in the planning and interpretation of SAND experiments where a strongly three-dimensional distribution of scattering intensity is expected, *GRIP* also includes a calculator. This tool can convert between neutron wavelength in ångströms, energy in meV and momentum in inverse ångströms. This calculator can also calculate reciprocal lattice vectors given lattice parameters (only necessary for conversions using RLU), and convert a Cartesian 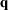 vector into RLU coordinates or the rotation angles at which a diffraction peak may appear. Note that for the conversion to angles one angle of {*san*, 2θ, ϕ, ψ} must be specified; otherwise the nonlinear function solver will be underconstrained. If given RLU coordinates, the calculator can convert those to Cartesian 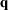
coordinates. Given a set of instrument angles, a corresponding 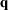 vector can also be calculated.

### 2.2. Binning

In a standard SAND experiment on samples with diffraction peaks, the sample is rocked along one or more angles while collecting data on a 2D area detector, sweeping out a 3D volume of reciprocal space. The pixels are not distributed evenly or uniformly in three dimensions, and therefore it is necessary to rebin the pixelated data into a regular coordinate system in order to plot the data for 1D, 2D or 3D cuts. In the following, we describe the protocol for binning in Cartesian coordinates. The procedure is similar for other coordinate systems, though care must be taken to handle the branch cut in the azimuthal angle in cylindrical and spherical coordinates if the desired limits exceed the range [0°, 360°]. In this case, the ψ coordinates should first be shifted as ψ → mod(ψ − ψ_min_, 360°) + ψ_min_ to ensure that the desired range is covered, where ψ_min_ is the minimum of the binned ψ range. To bin the data, first the user selects 0, 1 or 2 axes to integrate over for 3D, 2D and 1D plots, respectively. Then the range and number of bins of 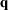-space to include is specified with minima and maxima in *q*_*x*_, *q*_*y*_ and *q*_*z*_. If the user leaves an intensity limit as NaN (‘not a number’), the largest/smallest value on that axis within the dataset is used automatically. The specified number of bins are evenly distributed between the minimum and the maximum. For each detector pixel at each sample angle, the pixel 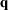 is calculated and is added to the appropriate bin for which it falls within the binning limits. Axes that are integrated over include all counts with the specified limits (this can be thought of as creating one bin for that axis). If normalization is selected, the counts in each bin are divided by the number of detector pixels that fell within that bin. This compensates for the nonuniform sampling of each bin (*e.g.* the Lorentz factor and moiré patterns between detector pixels and bins). Even with normalization, some choices of binning still lead to obvious binning artifacts, and care must be taken when aiming to evaluate peak intensities on an absolute scale from binned data.

**Figure 3:**
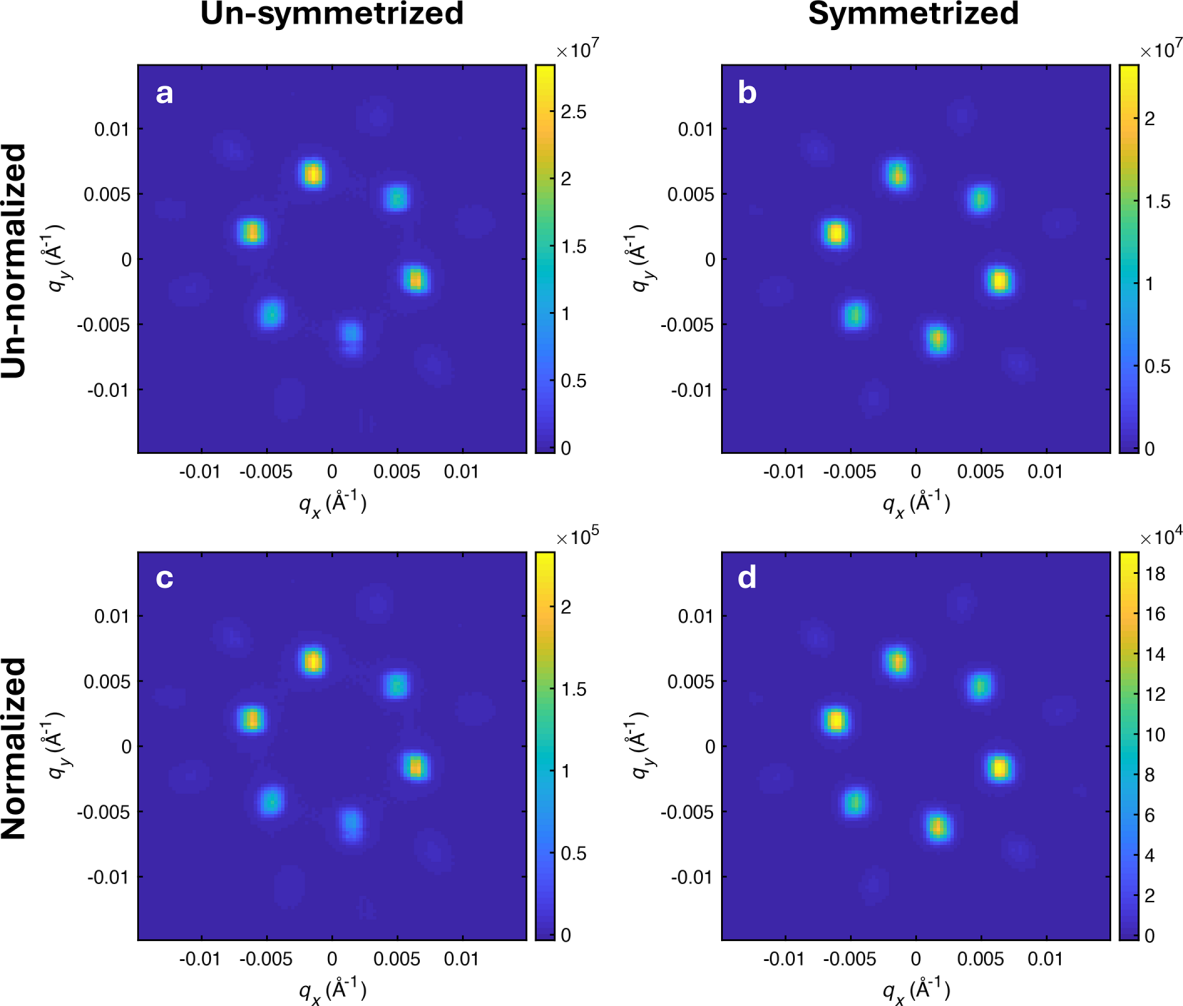
Comparison between options for data symmetrization and normalization within *GRIP*. The same data are plotted in all plots integrating over *q*_*z*_ with different symmetrization/normalization options. The two top plots (*a*, *b*) do not normalize the number of pixels per bin, while the two bottom plots (*c*, *d*) do. The two left plots (*a*, *c*) do not symmetrize the data 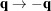, while the two right plots (*b*, *d*) do. The data are from the superconducting vortex lattice of elemental niobium measured on the SANS-I instrument, Paul Scherrer Institute, Switzerland.

The user can also choose to symmetrize the data with respect to 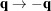. This is often valid because of Friedel’s law, which states that the intensity of scattering should be inversion symmetric because it is proportional to the magnitude squared of the structure factor. However, we note that data symmetrization may not always be appropriate due to absorption, an asymmetric background, detector inefficiency, the chiral term in polarized data *etc*. Whether or not to symmetrize should be judged carefully by the user on the basis of the details of their experiment and reported in publications. Symmetrization is useful to improve the statistics in data visualization and to accommodate for regions that were not reached in 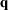-space. The symmetrization is performed by splitting each pixel of intensity *I* at position 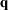 into two pixels of intensity *I*/2 at 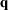 and 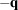. A comparison between a single *q*_*x*_–*q*_*y*_ cut with different choices of normalization and symmetrization is provided in Fig. 3 ▸. Symmetrization matches the intensity of opposing peaks and improves signal to noise. Normalization can even out the peak intensities somewhat, correcting for *e.g.* the Lorentz factor, which in this case leads to an intensity equalization so that the top and bottom peaks appear more intense. In practice, normalization may not always help with Lorentz factor corrections, depending on the angular extent over which the measurements are performed.

## 3. Plotting

A number of plotting options have been integrated into *GRIP* to aid in interpretation, investigation and analysis of SAND data. The data plotted and analyzed by *GRIP* come from the dataset that is currently in the *GRASP* dataloaders and can be perused angle by angle on the main display. Therefore, any background subtractions, renormalizations, polarization analysis *etc.* done by *GRASP* can be easily transported into *GRIP* for further analysis. Multi-detector support is implemented, and as mentioned above, any *GRASP*-supported instrument can be made compatible with *GRIP* through the implementation of GRIP_san, GRIP_dom and GRIP_phi angles (as defined in Fig. 2 ▸) in the instrument configuration file. This must be done individually for each instrument to accommodate differences in naming and sign conventions for each instrument’s goniometer(s).

### 3.1. 3D plotting

#### 3.1.1. Isosurface and volumetric plots

**Figure 4:**
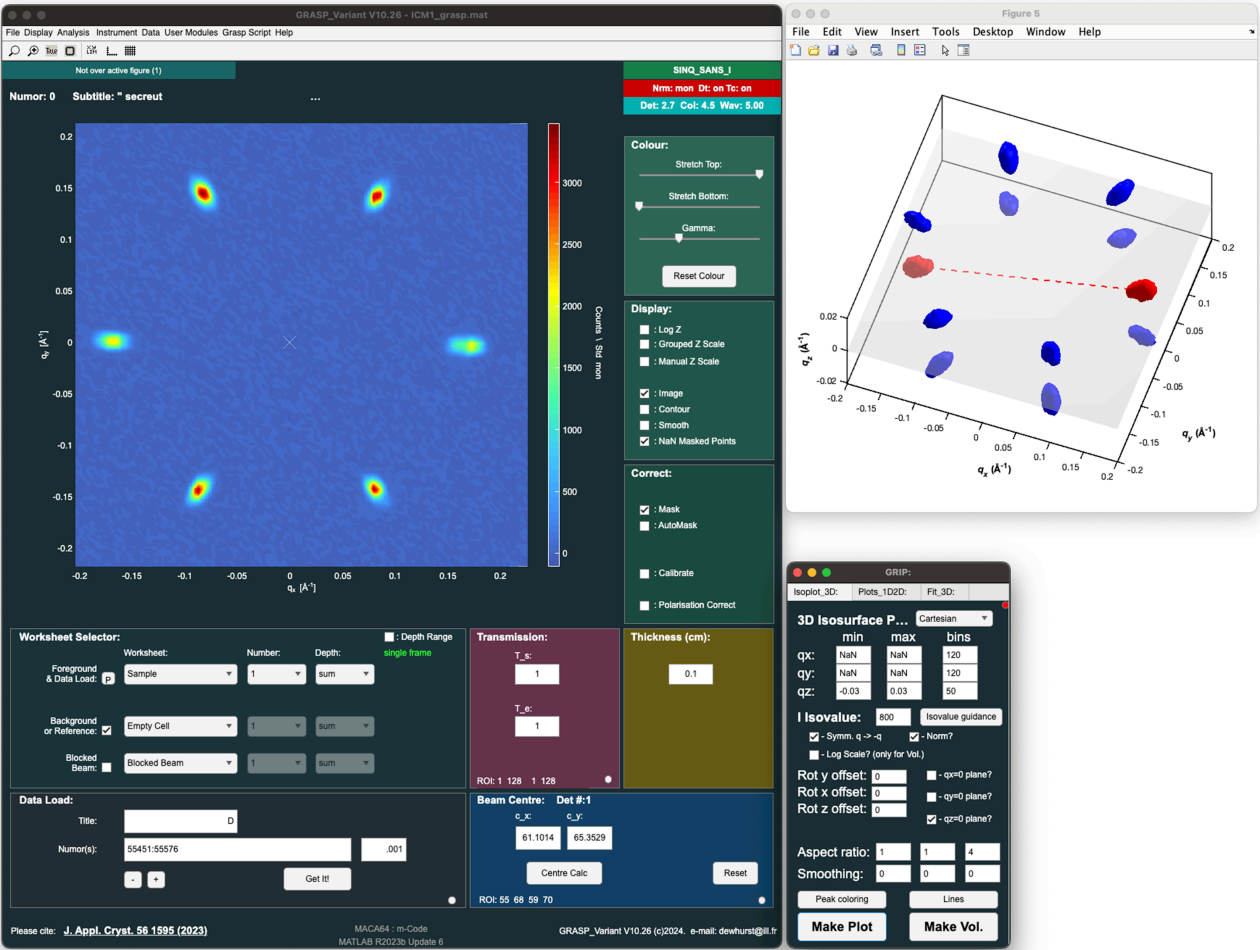
Example screenshot of main *GRASP* interface with data loaded from SANS-I (left), *GRIP* 3D plotting interface (lower right) and *GRIP* isosurface plot (upper right) for example data of an incommensurate magnetic material measured on the SANS-I instrument, Paul Scherrer Institute, Switzerland (Kurumaji *et al.*, 2024 ▸).

For full visualization of three-dimensional SAND datasets, *GRIP* includes a variety of 3D plotting options. Volumetric and isosurface plots in Cartesian and RLU coordinates can be made in the same GUI. First, the user selects the desired binning and symmetry/normalization settings. For the isosurface plotter, which plots three-dimensional isosurfaces of intensity, the user must specify the isovalue level below which the intensity is cut off. An isovalue guidance button is provided which gives the percentiles of binned pixel intensities. Often an isovalue near 99% is optimal to display the diffraction peaks, but some experimentation may be necessary to optimize the plot. For the isosurface plot, the user can also add translucent planes for *q*_*x*_ = 0, *q*_*y*_ = 0 and *q*_*z*_ = 0, as well as colored lines and surface coloring, as is shown in Fig. 4 ▸ for data of an incommensurate magnetic material measured on the SANS-I instrument, Paul Scherrer Institute, Switzerland (Kurumaji *et al.*, 2024 ▸).

**Figure 5:**
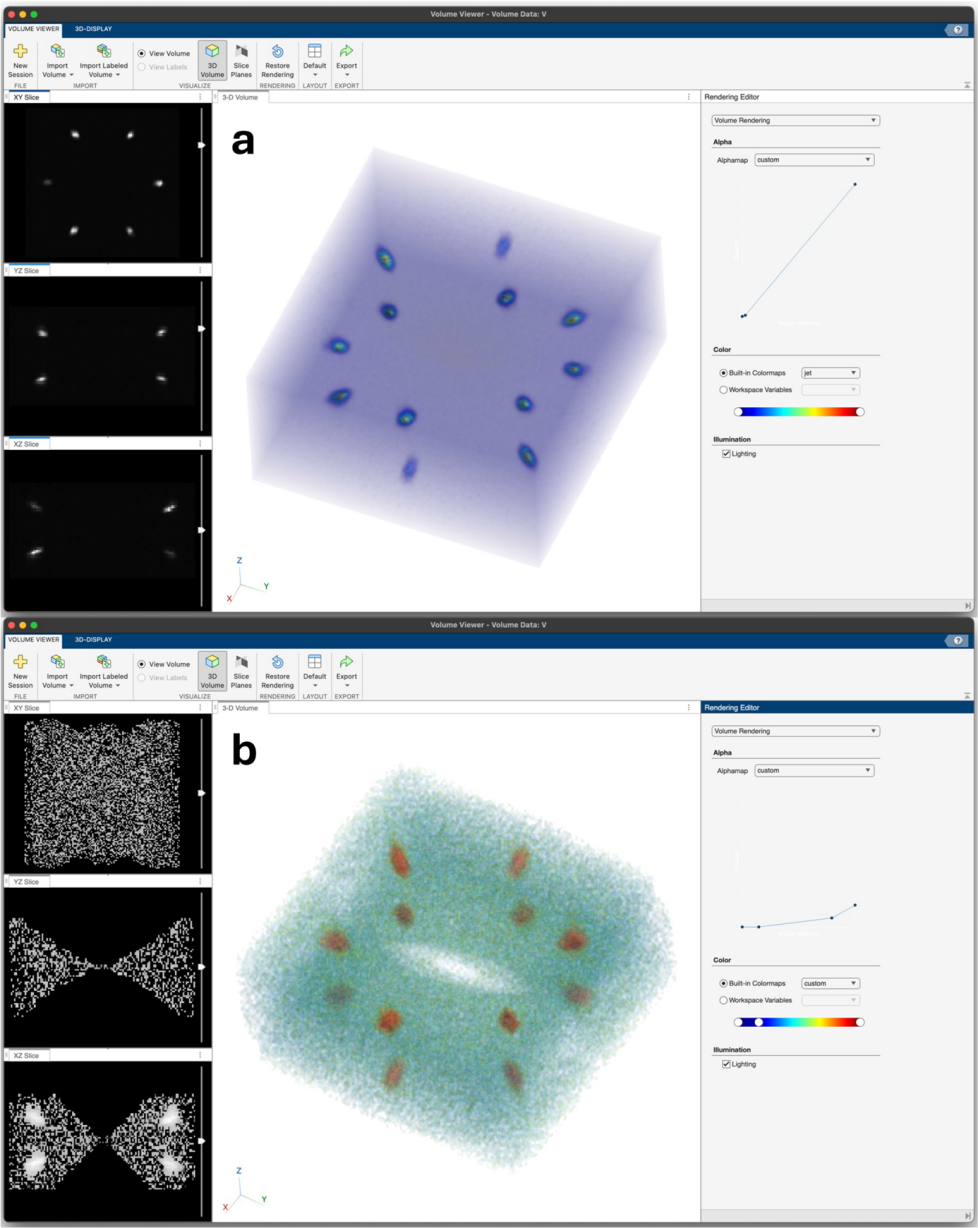
Example volumetric plots. (*a*) A linear-scaled plot of the main detector and (*b*) the same data with a logarithmic scale.

Volumetric plots where the opacity and color of a cuboid are modified by the scattering intensity can also be produced within MATLAB’s *Volume Viewer* interface. Again, first binning and symmetrization/normalization is specified. Volumetric plots can also be specified to be displayed with a logarithmic or linear intensity scale. When the ‘Make Vol.’ button is pressed, a new *Volume Viewer* window is created, and the user can choose a colorscale and manipulate the opacity scaling. The upper window shown in Fig. 5 ▸ includes a zoom-in of a linear scaled dataset, while the lower portion of Fig. 5 ▸ provides a logarithmic volumetric plot of the same dataset, but zoomed out to also show the volume mapped out by additional side-detector panels of the instrument. Both scales can be useful to highlight different aspects of the data. Additionally, in our experience the volumetric plotter is often the easiest way to discover subtle, inherently 3D features in the data, such as subtle three-dimensional peak shapes, mosaicity and harmonic peaks. The eye can detect these patterns across multiple voxels in three dimensions when using the volumetric plotter, which is not possible when the data are projected into two or one dimension. Note that this plotting option requires the MATLAB *Image Processing Toolbox* to be installed.

Both the volumetric and isosurface plotters include an anisotropic 3D Gaussian smoothing option and an aspect ratio option to adjust the relative scale of each axis. In particular, enlarging the scale of *q*_*z*_ is helpful when examining data from SAND experiments where the intensity is largely located near the *q*_*x*_-*q*_*y*_ plane.

#### 3.1.2. Pixel plotter

**Figure 6:**
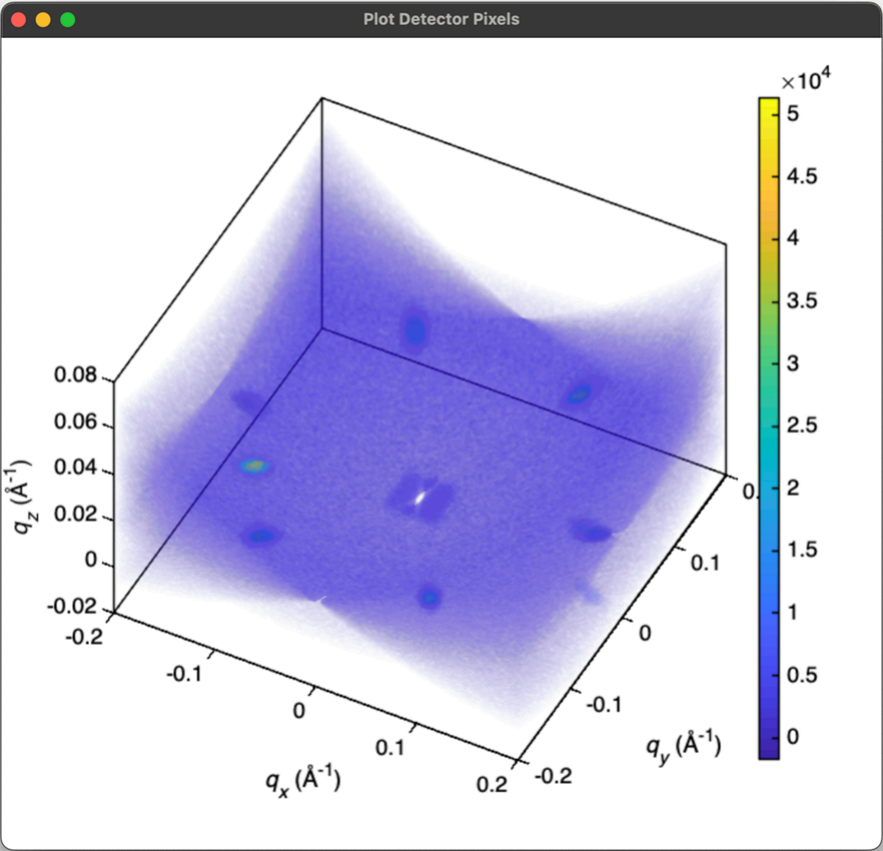
Example 3D pixel plot. Every pixel of each detector is plotted individually in the sample’s 3D reciprocal space for each angle in a rocking curve. The opacity and color of each pixel is scaled by its intensity.

Though the isosurface and volumetric plotters plot the binned data only, it may sometimes be useful to directly visualize the intensity distribution of the actually measured detector pixels (*i.e.* with no binning) in three-dimensional space. The 3D pixel plotter option enables this. Every exposure of each detector panel is plotted, curved into Cartesian momentum space. This is helpful to understand the region of momentum space sampled by a given rocking curve. The pixels can be given a uniform transparency value, or the transparency of each pixel can be scaled by its intensity. An example pixel plot is shown in Fig. 6 ▸.

### 3.2. 1D/2D plotting

**Figure 7:**
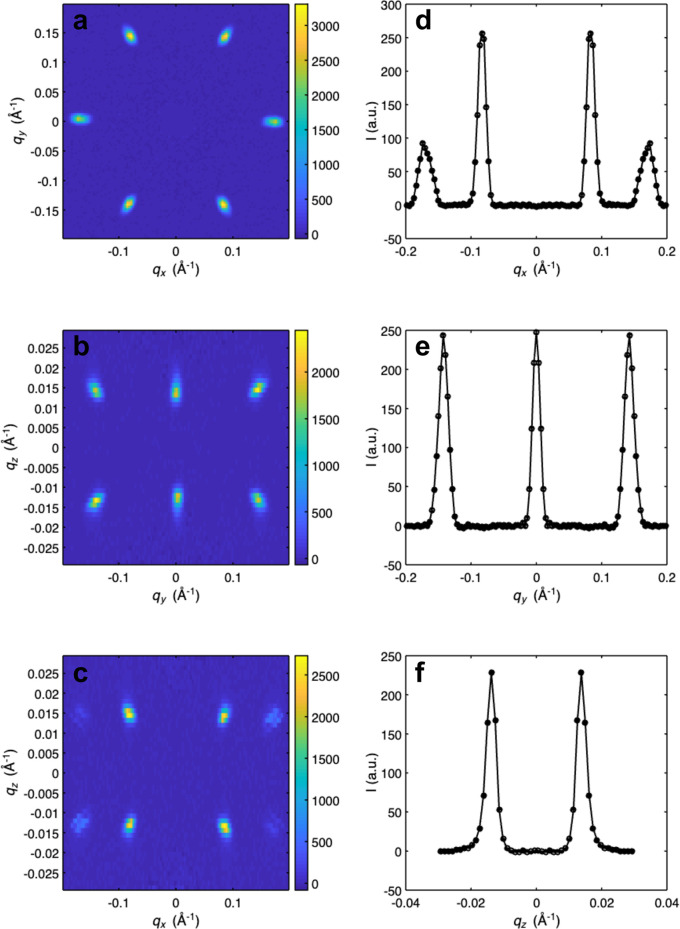
Screenshot of *GRIP* Cartesian 1D/2D multiplot viewer. Multiplot viewers of additional coordinate systems are provided in Appendix *A*[App appa]. (*a*) *q*_*x*_–*q*_*y*_ 2D colorplot with dataset integrated over *q*_*z*_. (*b*) *q*_*y*_–*q*_*z*_ 2D colorplot with dataset integrated over *q*_*x*_. (*c*) *q*_*x*_–*q*_*z*_ 2D colorplot with dataset integrated over *q*_*y*_. (*d*) *q*_*x*_ 1D lineplot with dataset integrated over *q*_*y*_ and *q*_*z*_. (*e*) *q*_*y*_ 1D lineplot with dataset integrated over *q*_*x*_ and *q*_*z*_. (*f*) *q*_*z*_ 1D lineplot with dataset integrated over *q*_*x*_ and *q*_*y*_.

In addition to 3D plotting, 1D and 2D plots can be produced in *GRIP*. These are often useful for a subsequent, more detailed quantitative analysis. The user can choose either to make a single 1D or 2D plot or to make a multiplot which includes six 2D and 1D cuts of the same dataset, as is shown in Fig. 7 ▸ and in Appendix *A*[App appa] in Figs. 10, 11 and 12. These plots can be produced in Cartesian, cylindrical, spherical and RLU coordinates. The user must specify the coordinate system, the binning and which axes to integrate over, the symmetrization/normalization options, whether to plot in a logarithmic scale, and the colorscale. When using the multiplotter, the axes of all six plots can be linked together, allowing the user to zoom in to specific features. Then the limits can be transferred back to the 1D/2D plotting GUI or peak fitting GUI (discussed below) to easily plot only a specific volume of reciprocal space.

## 4. 3D peak fitting and resolution function

**Figure 8:**
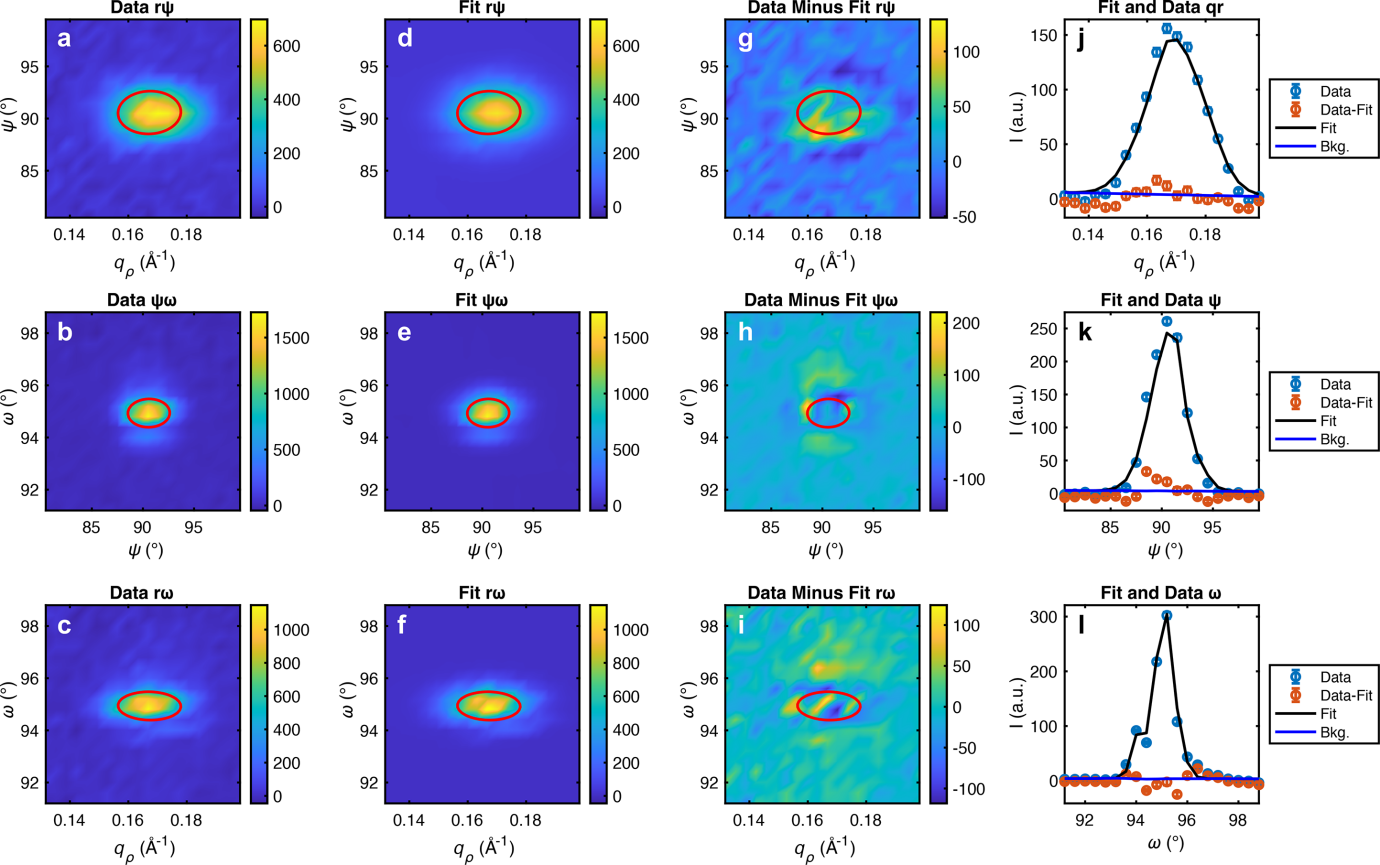
Screenshot of 3D peak fitter. (*a*–*c*) Data binned into 2D colorplots on the indicated axes, with one axis integrated in each. (*d*–*f*) Fit binned in the same way into 2D colorplots. (*g*–*i*) Residuals of data minus fit. The red ovals indicate the one standard deviation contour of the Gaussian ellipsoid fitting function.(*j*–*l*) 1D lineplots with the data binned to *q*_ρ_, ψ and ω respectively, each integrated over the other two axes. The data are shown with blue circles, the residuals with red circles, the fit with a black line and the linear background with a blue line. The error bars shown are statistical. The fit parameters are output to the terminal.

To more accurately obtain peak positions, heights and widths for quantitative analysis, *GRIP* includes functionality to fit a diffraction peak to a 3D Gaussian ellipsoid with a linear background function: 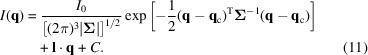
Here 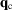 is the peak center, 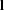 is a vector of linear background coefficients, *C* is a uniform background offset and **Σ** is a covariance matrix: 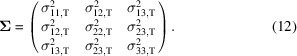
The fitting can be done in any coordinate system, so {1, 2, 3} correspond to the first, second and third dimensions in the coordinate system of choice. The covariance 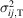 quantifies how correlated the peak shape is along axes *i* and *j*, with σ_*ii*,T_ being the variance (square of the standard deviation) along axis *i*. First, the user should select a volume of momentum space which only contains one peak. This can be done quickly by linking the axes of the multiplotter, zooming in to the desired region and pressing ‘Get Lims’ in the fitting GUI. Then the user can choose to symmetrize the data and apply any angular misalignment offset. The peak fitter automatically guesses the initial fit parameters and performs a nonlinear least squares fit of equation (11) directly to all detector pixels within the selected limits, not the binned data. The number of bins selected does not affect the fitting result and is only used in the fitting result visualization as shown in Fig. 8 ▸. This is why some fit results may not look like smooth Gaussians when binned.

**Figure 9:**
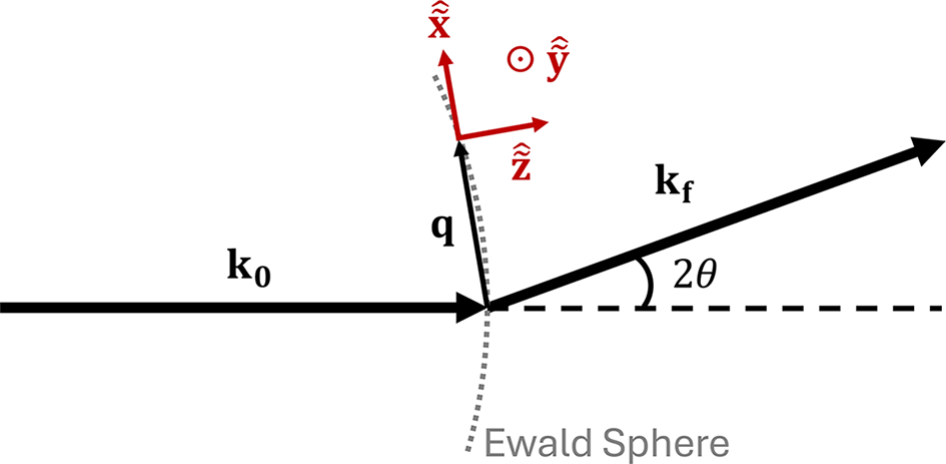
Coordinate system used in defining the principal axes of the SANS resolution function.

**Figure 10:**
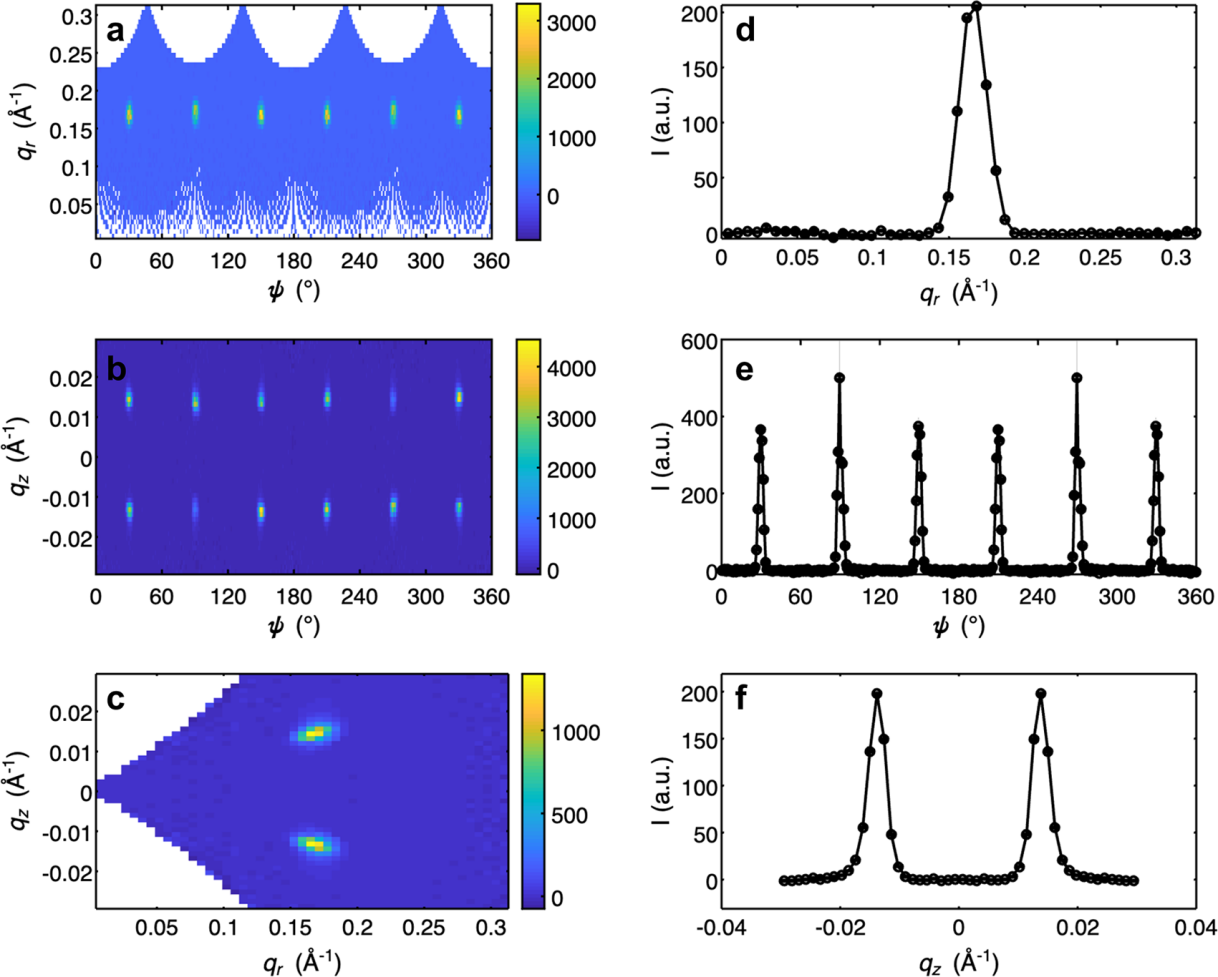
Screenshot of *GRIP* cylindrical 1D/2D multiplot viewer. (*a*) ψ–*q*_*r*_ 2D colorplot with dataset integrated over *q*_*z*_. (*b*) ψ–*q*_*z*_ 2D colorplot with dataset integrated over *q*_*r*_. (*c*) *q*_*r*_–*q*_*z*_ 2D colorplot with dataset integrated over ψ. (*d*) *q*_*r*_ 1D lineplot with dataset integrated over ψ and *q*_*z*_. (*e*) ψ 1D lineplot with dataset integrated over *q*_*r*_ and *q*_*z*_. (*f*) *q*_*z*_ 1D lineplot with dataset integrated over *q*_*r*_ and ψ.

**Figure 11:**
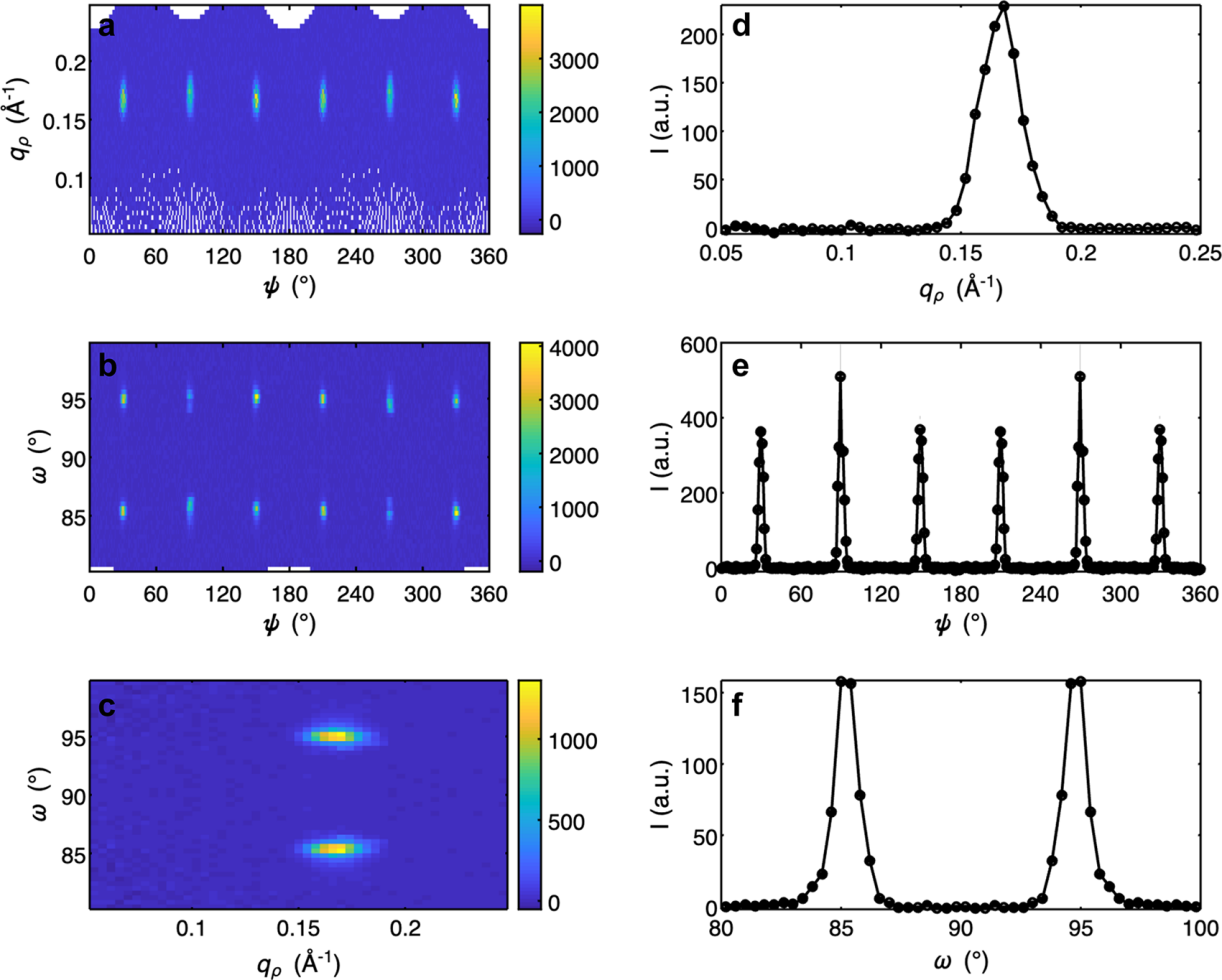
Screenshot of *GRIP* spherical 1D/2D multiplot viewer. (*a*) ψ–*q*_ρ_ 2D colorplot with dataset integrated over ω. (*b*) ψ–ω 2D colorplot with dataset integrated over *q*_ρ_. (*c*) *q*_ρ_–ω 2D colorplot with dataset integrated over ψ. (*d*) *q*_ρ_ 1D lineplot with dataset integrated over ψ and ω. (*e*) ψ 1D lineplot with dataset integrated over *q*_ρ_ and ω. (*f*) ω 1D lineplot with dataset integrated over *q*_ρ_ and ψ.

**Figure 12:**
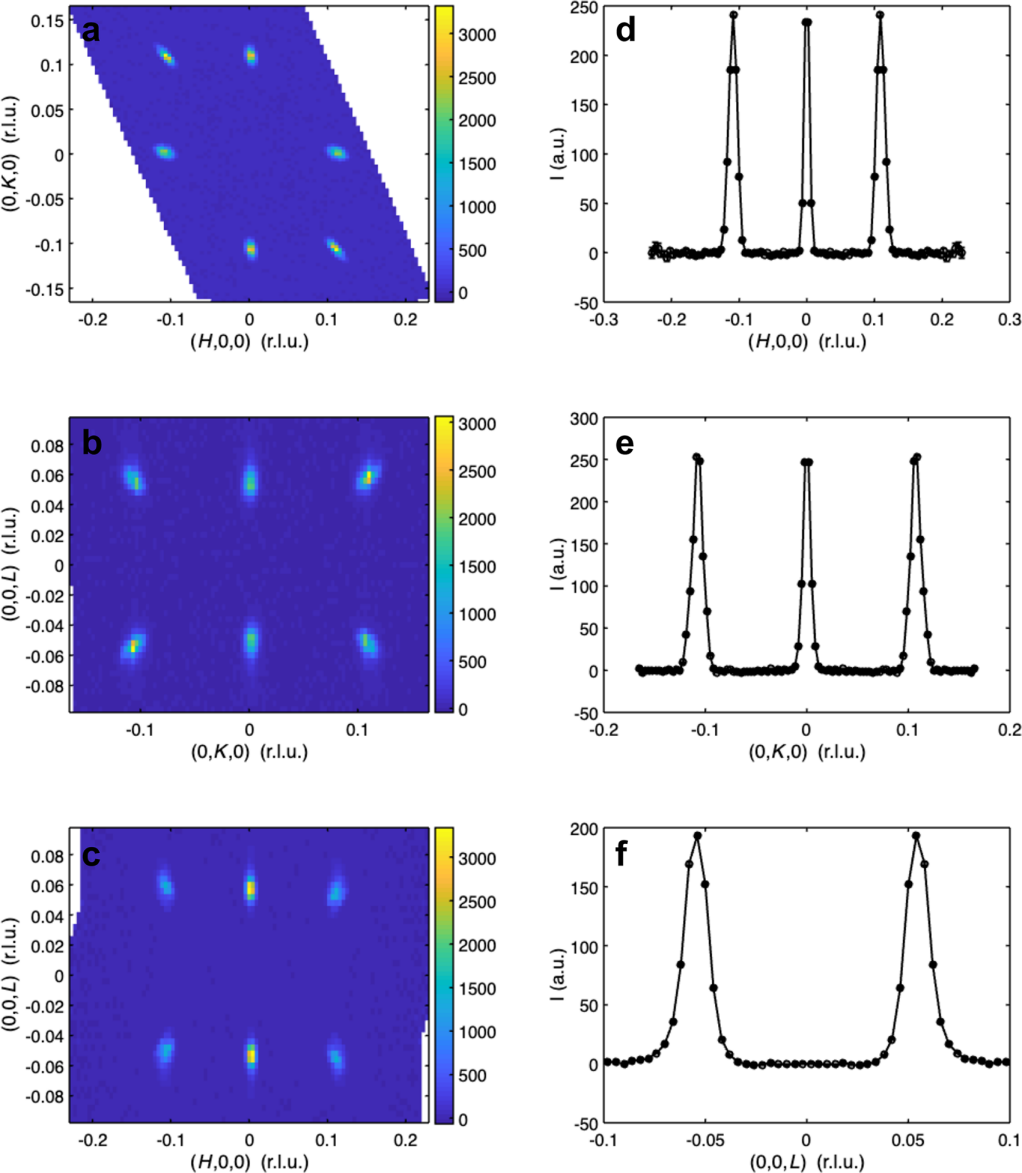
Screenshot of *GRIP* RLU 1D/2D multiplot viewer. (*a*) *H*–*K* 2D colorplot with dataset integrated over *L*. (*b*) *K*–*L* 2D colorplot with dataset integrated over *H*. (*c*) *H*–*L* 2D colorplot with dataset integrated over *K*. (*d*) *H* 1D lineplot with dataset integrated over *K* and *L*. (*e*) *K* 1D lineplot with dataset integrated over *H* and *L*. (*f*) *L* 1D lineplot with dataset integrated over *H* and *K*.

One of the key advantages of SAND measurements is that they can be used to obtain information about sample correlation lengths in all three dimensions from the widths of diffraction peaks (Pedersen *et al.*, 1990 ▸; Harris *et al.*, 1995 ▸). If a peak has total width (standard deviation) σ_*ii*,T_ along axis *i*, this is due to a convolution of the instrument resolution in that direction, σ_*ii*,I_, with the intrinsic width of the peak from the sample, σ_*ii*,S_, such that 
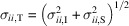
. If σ_*ii*,S_ is provided in units of Å^−1^ or nm^−1^, then the sample correlation length in that direction is given by λ_*i*_ = 1/σ_*ii*,S_ in units of Å or nm. The standard deviations σ can be converted to a full width at half-maximum (FWHM) by multiplying by 2(2ln2)^1/2^ ≈ 2.355, and similarly the FWHM correlation length is obtained by dividing λ_*i*_ by 2(2ln2)^1/2^. The principal axes of the SAND resolution function lie approximately along the red axes depicted in Fig. 9 ▸: one along 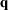, one perpendicular to the surface of the Ewald sphere (we note that the lineshape in this direction is often of more Lorentzian or Voigt character) and a third orthogonal to those. *GRIP* implements several methods of estimation of the instrument resolution, which are discussed in detail in Appendix *B*[App appb]. The results of the calculations by these methods can be compared with one another by the user and provide a sense of the uncertainty in estimations of the instrument resolution.

Once the instrument configuration is input into the instrument resolution estimator, the results can be combined with the 3D peak fitter to obtain an estimate of the correlation length of a sample in all three directions. This is most easily performed with a peak fit in spherical coordinates, as a standard SANS instrument will possess an instrument resolution ellipsoid whose principal axes are along *q*_ρ_, ψ and ω which are locally parallel to the red axes in Fig. 9 ▸.

## 5. General guidance for a SAND experiment

Generally, little needs to be changed in a SAND experiment from a conventional SANS experiment on three-dimensional structures. Similar collimation and sample-to-detector selection choices apply. The greatest potential differences lie in the choice of rocking angle step size, range and axes. For the best results, the rocking axes and range should be selected to completely rock through each feature of interest at least once. At the extreme, a full 180° rocking curve (or 360° if the detector is offset from the beam center) covers the full volume of reciprocal space. Then the step size should be chosen to take multiple steps to pass through each peak, and ideally should be less than or equal to the instrument resolution in ω at the wavevectors of interest. Neglecting considerations of overhead for motors to change *etc.*, sampling with denser rocking curve steps for less time each is strictly better for *GRIP*, because these data can always be rebinned to a courser grid. If the step size is too large, some binned voxels will contain no detector pixels and will be blank in the cuts unless a very course binning is chosen. Exploitation of the *GRIP* resolution estimator allows the operator to estimate reasonable rocking curve step sizes when planning SANS intensity mapping measurements.

## 6. Conclusion

Here, we have described the implementation of a 3D SANS plotting and analysis package called *GRASP Integrated 3D Plotter* (*GRIP*), which is integrated into the *GRASP* program (Dewhurst, 2023 ▸). The *GRIP* module enables SANS users to easily observe three-dimensional scattering information in near real time during a beamline experiment and plot and analyze the data in detail after the experiment. The *GRIP* module significantly extends the capabilities of *GRASP*, enabling a more comprehensive analysis of 3D intensity distributions collected using SANS instruments. We envisage the module as being a routinely used tool for analyzing data from intrinsically three-dimensional volume datasets, such as incommensurate spin textures, SANS tomographic measurements (Henderson *et al.*, 2023 ▸) *etc*.

The presently described *GRIP* module is developed for handling data obtained in monochromatic SAND experiments, as well as for Gaussian fitting of volumetric data. Our approach is not limited to these cases however, and extensions to the *GRIP* module can be easily implemented to include new features. For example, routes for further development include more peak fitting options catering for non-Gaussian functions, 1D and 2D fitting, dealing with overlapping/multiple peaks, numerical approaches to resolution function calculations (*e.g.* with Monte Carlo methods), compatibility with scripting, more flexible goniometer setups, additional ways to cut the data, implementation of a generalized Bayesian analysis approach (Holmes, 2014 ▸) to 3D volumetric data, and handling data obtained in time-of-flight modes. New instruments or detector upgrades can be easily accommodated, as *GRIP* is built within *GRASP*, which is built to flexibly handle a variety of instruments.

## Appendix A Multiplots in additional coordinate systems

The data plotted in Fig. 7 ▸ can also be plotted in other coordinate systems as defined in Fig. 2 ▸. Figs. 10 ▸, 11 ▸ and 12 ▸ provide screenshots of the same data plotted in cylindrical, spherical and RLU coordinates, respectively.

## Appendix B Approaches to calculating the instrument resolution

Several methods of resolution estimation are implemented in *GRIP*. In the following, a quantity with angled brackets 〈…〉 indicates the nominal value, while a quantity without signifies the true value. Quantities with Δ indicate a FWHM while quantities with σ indicate a standard deviation. Consider an instrument with FWHM wavelength spread Δλ/〈λ〉 (typically ∼0.1), a first aperture width and height *w*_*x*1_ and *w*_*y*1_, respectively, a collimation length (distance from first aperture to sample) *l*_1_, a second aperture or sample size, whichever is smaller, width and height *w*_*x*2_ and *w*_*y*2_, respectively, a sample-to-detector distance *l*_2_, and pixel size *w*_*x*3_ by *w*_*y*3_. The following equations assume circular collimating apertures; if rectangular apertures are used, then *w*_*x*1_, *w*_*y*1_, *w*_*x*2_ and/or *w*_*y*2_ should be multiplied by 2(3^1/2^)/3. We consider the resolution in ,  and  along the red axes in Fig. 9 ▸ for nominal scattering vector magnitude 〈*q*〉.

### b1. Method 1: Taylor expansion

One can consider the reported wavevector 〈〉 and the actual wavevector  as a function of the expected and true values, respectively, of the neutron flight path through the SANS instrument. First, the neutron has a wavelength λ. Then, the vertical and horizontal position where the neutron entered the first collimating aperture is (*x*_1_, *y*_1_). Similarly we have (*x*_2_, *y*_2_) for the second aperture, and the position on the detector (*x*_3_, *y*_3_). Each of these parameters has a nominal value 〈*x*_1_〉 = 〈*x*_2_〉 = 〈*y*_1_〉 = 〈*y*_2_〉 = 0, 〈*x*_3_〉, 〈*y*_3_〉 and 〈λ〉, as well as an uncertainty σ_*xi*_ = *w*_*xi*_ / 4, σ_*yi*_ = *w*_*yi*_ / 4 and  = . If the aperture is circular instead of rectangular, the 4 should be replaced with 12^1/2^.  can be written in the laboratory Cartesian coordinate system as a function of these parameters as

Inserting the nominal values of these parameters, we obtain

with *k*_0_ = 2π/〈λ〉 and , which is equivalent to equation (1). We can then transform into the spherical/principal resolution function coordinate system and propagate the uncertainty in  as

where  is a shorthand meaning to evaluate with the nominal values of all parameters. This propagation is not concise but can easily be performed exactly with symbolic computations in MATLAB. This is a first-order estimate of the uncertainty in , assuming each parameter is independent.

### b2. Method 2: Monte Carlo

We can use a simple Monte Carlo approach to simulate the instrument resolution function. In addition to all above values, we include the range and step size of rocking of GRIP_san. For each neutron, a wavelength is chosen randomly, distributed as a Gaussian/triangle distribution about 〈λ〉 with the correct FWHM. Next, a random position on each of the first and second apertures is chosen under the assumption that both apertures are uniformly illuminated. For the momentum vector  under consideration, it is determined which value, if any, of GRIP_san satisfies the Bragg condition for this incident neutron. Then, the outgoing neutron direction is calculated and used to obtain the location where this neutron will hit the detector. The four possible solutions for GRIP_san are

with

where the valid solutions can be identified by confirming that . The location where the neutron hits the detector and the value of GRIP_san are both binned into the specified binning mesh. The standard deviation along each direction is then calculated. This approach allows one to simulate how the data would look for a given rocking curve and even permits the data to be exported back to *GRASP* for further analysis and comparison.

### b3. Method 3: estimation

The SANS instrument resolution function can be estimated (Pedersen *et al.*, 1990 ▸; Harris *et al.*, 1995 ▸) as originating from three sources: the wavelength spread, the angular collimation from the collimating apertures and the detector resolution. The following assume that  lies in the horizontal scattering plane; for scattering out of the plane, *w*_*xi*_ and *w*_*yi*_ must be appropriately mixed.

The wavelength spread only affects the resolution along . This contribution is

The collimation affects all three components as

with *k*_0_ = 2π/〈λ〉 and θ = arcsin(〈q〉/2*k*_0_). Δβ_1_ and Δβ_2_ are given by

and

where *a*_1_ = *w*_*x*1_/[2(*l*_1_ + *l*_2_)] and *a*_2_ = *w*_*x*2_/(2*l*_2_). Finally, the detector resolution is

These are all added in quadrature to obtain the complete instrument resolution along all three axes as

### b4. Method 4: semi-empirical

The angular collimation of the beam and detector resolution can be directly measured by measuring the size of the (attenuated) direct beam on the detector. This has the advantage of including all effects of divergence without any assumptions of perfect optics alignment *etc*. The following analysis is valid for small angles (). If the direct beam is measured to have an FWHM size *w*_D_ on the detector, then the beam has an effective angular spread of

The resolution along *q*_*x*_ is then approximately

the *q*_*y*_ component is

and the *q*_*z*_ component is
